# Knowledge and attitudes regarding elective oocyte cryopreservation in undergraduate and medical students

**DOI:** 10.1186/s40738-019-0057-9

**Published:** 2019-04-10

**Authors:** Arnold M. Mahesan, Seifeldin Sadek, Hadi Ramadan, Silvina Bocca, Anthea B. M. Paul, Laurel Stadtmauer

**Affiliations:** 1 Jones Institute for Reproductive Medicine, Eastern Virginia Medical School, 700 W Olney Rd, Norfolk, VA 23507 USA; 2 University of Ottawa, Ottawa, ON Canada

**Keywords:** Elective oocyte cryopreservation, Social fertility preservation, Undergraduates, Medical students

## Abstract

**Background:**

To assess knowledge and attitudes regarding elective oocyte cryopreservation among female undergraduate students (UG) and medical students (MS) in Eastern Virginia.

**Methods:**

An anonymous cross-sectional study surveying female UG at a local university and MS at our academic medical center in May of 2017. The survey contained questions on demographic information, interest in fertility preservation, and knowledge about age related changes in fertility.

**Results:**

There were 74 of 102 female UG and 95 of 117 female MS who responded, for a response rate of 73 and 81% respectively. UG were significantly younger than MS (21.4 vs 26.8, *p* < 0.001). Further, UG generally planned on conceiving at a younger age than MS (age 26–30 vs 31–35), and favored younger ages to consider oocyte cryopreservation (age 26–30 vs 31–35). Only a minority of both UG and MS were willing to undergo egg freezing at the current price of approximately $10,000 (15% vs 26% respectively, *p* = 0.044). Moreover, 73% of students overall responded that they would be more likely to freeze oocytes if their employer paid. Notably, both UG and MS underestimated age of fertility decline.

**Conclusion:**

Both UG and MS revealed a need for education on age-related changes in fertility. Most UG and MS would not undergo elective oocyte cryopreservation at the present cost but would consider it at a lower cost.

**Electronic supplementary material:**

The online version of this article (10.1186/s40738-019-0057-9) contains supplementary material, which is available to authorized users.

## Background

The average age of first-time mothers in the United States increased by 1.4 years from 2000 to 2014, from 24.9 years to 26.3 years [1]. With improvement in assisted reproductive technology (ART) more women are seeking infertility treatment at advanced maternal ages. In the United States in 2015, 12,943 embryo transfers were performed in women aged 35–37 years, and 18,429 were performed in women aged > 37 years [2, 3].

The delay in childbearing is thought to stem from social and economic factors [4]. With the development of oocyte cryopreservation and the vitrification process, women who desire to postpone pregnancy can avoid the need for donor oocytes and can use their own eggs at a later age [5]. Both egg freezing and egg donation are costly, however technologies that allow preservation of fertility until later in life have increasingly entered the public eye [6, 7]. The strong demand for these technologies is reflected by the recent offering of oocyte cryopreservation costs as an employee benefit by prominent companies such as Facebook and Apple [6].

Knowledge on elective oocyte freezing varies widely, even amongst physicians [7]. In a study of 410 Israeli undergraduate students, students overestimated women’s chances of spontaneous pregnancy in all age groups. Only 11% of students knew that genetic motherhood is unlikely to be achieved from mid-40s onwards, unless using oocytes frozen in advance or donor oocytes from a younger donor. [8, 9]. In another study of 328 university students in Northern California, 79% were interested in learning about the current status of ovarian reserve, but only 29% would consider stopping professional pursuits to focus on conceiving [10].

UG and MS are at a stage in their lives where they are considering their future and career goals. Given the currently increased visibility and feasibility of elective oocyte cryopreservation technology, these students are increasingly faced with oocyte cryopreservation as an option that may influence their life and career decisions. We conducted the present study to explore knowledge and attitudes regarding elective oocyte cryopreservation among female undergraduate students and medical students in Eastern Virginia.

## Materials and methods

We conducted an anonymous cross-sectional study surveying female undergraduate students at a local university and medical students at our academic medical center in December of 2017. Printed questionnaires developed by the authors of the study were distributed to students at the end of classes and completion was voluntary and confidential. Undergraduate surveys were administered in introductory biology classes, and medical school surveys were administered in fourth year full-class didactic sessions. These sessions were on general issues regarding life after graduation, and no background reading was given to the students. Surveys were handed only to students who indicated they desired to complete them. These students returned the surveys anonymously to a box before they left the classroom. The survey contained questions on demographic information, interest in fertility preservation, and knowledge about age related changes in fertility. (Additional file 1) The study was approved by the Eastern Virginia Medical School Institutional Review Board (IRB #: 17·05-XX-0133).

Descriptive statistics for the answers to survey questions were calculated. Medical student responses were compared to undergraduate student responses. Continuous variables were compared using unpaired Student’s t-test, and categorical variables were compared with Chi-squared test using SPSS™ (SPSS Statistical Software V.22.0, IBM, Armonk, NY). *P* values of less than 0.05 were considered significant.

## Results

### Population characteristics

The characteristics of the population surveyed are presented in Table 1. One hundred sixty-nine of 219 female students chose to complete the survey, for an overall response rate of 77%. This include 74 of 102 female undergraduates and 95 of 117 female medical students, for group specific response rates of 73 and 81% respectively. The mean age of all participants was 24 +/− 4.0. The mean age of undergraduate students was 21.4 +/− 3.5, which was significantly younger than the mean age of medical students, 26.8 +/− 2.3 (*p* < 0.00001).

**Table 1:** Surveyed Population Characteristics

	Total (*n* = 169)	Undergraduates (*n* = 74)	Medical students (*n* = 95)	*p* value
Mean Age (SD)	24.0 (4.0)	21.6 (3.5)	26.8 (2.3)	< 0.00001
Race (%)				0.00027
White	55	46	62	
Hispanic/Latino	5	11	1	
African American	14	19	12	
Asian	17	11	22	
Other	7	12	3	
Religion (%)				0.011
Christian	37	39	36	
Catholic	13	15	13	
Non-religious	34	38	32	
Muslim	2	1	4	
Hindu/buddhist/other	11	3	18	
Have children (%)				0.74
Yes	4	4	5	
No	96	95	95	
Plan to have children (%)				0.0001
Yes	65	53	80	
No	20	27	8	
Unknown	15	20	12	
Age plan to have first child (%)				0.022
21–25	4	5	0	
26–30	30	41	21	
31–35	28	24	33	
35–40	2	0	4	
40+	1	0	1	
Unknown	33	27	39	
Feel pressure from family to have child (%)				0.46
Yes	26	28	24	
No	73	69	75	

Overall, 55% of participants were White, 17% were Asian, 15% were African American, and 5% were Hispanic/Latino. The most common religious denominations overall were Christian at 37% and non-religious at 34%. Medical students differed significantly from undergraduates in race, with medical students having higher proportions of White (62% vs 46%) and Asian (22% vs 11%) and lower proportions of African American (12% vs 19%) and Hispanic/Latino students (1% vs 11%), *p* = 0.00027. Medical students also different significantly in religion, with a larger proportion identifying as Hindu/Buddhist/other (18% vs 3%, *p* = 0.011).

Only 4% of participants surveyed had children and 64% overall planned to have children. Significantly more medical students than undergraduates planned to have children, 80% vs. 53% (*p* = 0.0001). For those who planned to have children, 33% of medical students planned on conceiving between age 31–35, whereas 40% of undergraduates planned on conceiving at the younger age of 26–30 (*p* = 0.022). Finally, 73% of participants overall felt no pressure from family to conceive or have children, with no significant difference between undergraduate and medical students.

### Personal decisions regarding oocyte cryopreservation

Responses to questions on personal decisions regarding oocyte cryopreservation are presented in Table 2. Regarding which age to consider oocyte cryopreservation, 41% of undergraduates favored age 26–30, whereas 48% of medical students favored the older age of 31–35 (*p* = 0.028). Sixty-two percent of students overall would consider oocyte cryopreservation for career reasons, 47% would consider it for medical reasons, and only 34% would consider it for social reasons, with no significant difference between medical students and undergraduates.

**Table 2:** Personal Decisions Regarding Elective Oocyte Cryopreservation

	Total (n = 169)	Undergraduates (n = 74)	Medical students (n = 95)	*p* value
Age would consider egg freezing				0.028
21–25	9	16	3	
26–30	28	41	19	
31–35	39	27	48	
36–40	8	5	11	
Above 40	1	1	1	
Would consider egg freezing for medical reasons				0.93
Yes	47	47	44	
No	53	51	49	
Would consider egg freezing for social reasons				0.62
Yes	34	32	35	
No	66	68	64	
Would consider egg freezing for career reasons				0.62
Yes	63	60	64	
No	37	39	36	
Would consider egg freezing at current price (~$10,000)				0.044
Yes	21	15	26	
No	20	19	20	
At lower price	57	66	46	
Who should pay for egg freezing				0.009
Self	38	42	34	
Insurance	52	55	50	
Employer	6	0	11	
If employer paid, would be more likely to freeze eggs				0.68
Yes	73	75	71	
No	26	25	27	

Undergraduates and medical students differed significantly in their willingness to undergo egg freezing at the current price (~$10,000), 15 and 26% respectively (*p* = 0.044). The majority of students, 52%, believed insurance should pay for oocyte cryopreservation and significantly more medical students, 11%, believed the employer should pay in comparison to 0% of undergraduates (*p* = 0.009). A large majority, 73% of students overall, believed that if an employer paid, they would be more likely to undergo oocyte cryopreservation.

We examined the data for significant correlations to other variables besides student type, and found that religion was associated with the decision to undergo oocyte cryopreservation. Seventy-nine percent of Christians and 76% of Non-religious participants believed that decreasing fertility with age would influence their decision to undergo oocyte cryopreservation. In comparison, only 48% of Catholics thought age would influence their decision (*p* = 0.039). Also, pressure from family to have a child was also significantly associated with the decision to cryopreserve, as 59% of participants who felt pressure thought they would consider oocyte cryopreservation compared with 41% who did not (*p* = 0.049).

### Knowledge about age-related changes in fertility

Table 3 presents responses to questions assessing knowledge about age-related changes in fertility. Significantly more medical students (68%) than undergraduates (59%; *p* = 0.032) believed they had some knowledge on these issues. When asked “At what age does natural fertility significantly decrease?”, 38% of undergraduates answered between ages 36–40 and 35% between ages 41–45, as compared with 62% of medical students who answered more correctly between ages 36–40 (*p* = 0.001).

**Table 3:** Knowledge about Age-Related Changes in Fertility

	Total (n = 169)	Undergraduates (n = 74)	Medical students (n = 95)	*p* value
Prior knowledge about egg freezing (%)				0.032
None	20	30	14	
Some knowledge	64	59	68	
Moderate knowledge	13	9	16	
Very knowledgeable	1	0	2	
At what age does fertility significantly decrease? (%)				0.001
Age 30–35	16	15	15	
Age 36–40	48	38	62	
Age 41–45	27	35	16	
Age 46–50	7	11	4	
What age is too old to have a child naturally? (%)				0.018
Age 30–35	2	4	0	
Age 36–40	9	8	9	
Age 41–45	44	32	48	
Age 46–50	41	53	32	
Does the fact of increased miscarriage risk with age influence your decision to freeze eggs? (%)				0.72
Yes	50	51	48	
No	50	49	51	
Does the increased risk of Down syndrome with age influence your decision to freeze eggs? (%)				0.039
Yes	54	47	59	
No	45	51	35	
What is the minimum number of frozen eggs at age 30 likely to result in a live birth? (%)				0.078
5–10 oocytes	18	11	21	
11–20 oocytes	38	34	40	
21–30 oocytes	26	34	19	
31–40 oocytes	8	9	7	
40+ oocytes	6	7	5	
What is the minimum number of frozen eggs at age 40 likely to result in a live birth? (%)				0.731
5–10 oocytes	4	5	7	
11–20 oocytes	12	9	13	
21–30 oocytes	30	26	32	
31–40 oocytes	25	27	22	
40+ oocytes	25	26	25	

55% of students who reported no prior knowledge and 62% of participants who reported moderate knowledge believed that age 46–50 was too old to have a child naturally, while 53% who claimed some (or little) prior knowledge thought age 41–45 was too old (*p* = 0.003). Further, 53% of undergraduates answered that age 46–50 is too old to have children naturally, while 48% of medical students answered age 41–45 (*p* = 0.018).

Most students, 72% overall, agreed that decreased fertility with age influences their decision to freeze oocytes. Students were split evenly on the increased risk of miscarriage with age influencing their decision: 50% answered “yes” with no significant difference between undergraduates and medical students. However, a significant difference emerged when asked about the influence of increased risk of having a child with Down Syndrome with age on their decision: 47% of undergraduates answered “yes” compared with 59% of medical students (*p* = 0.039).

When asked about the minimum number of frozen oocytes at age 30 or age 40 likely to result in a live birth, there was no significant difference between undergraduates and medical students. For age 30, 38% of students overall selected 11–20 oocytes. For age 40, 30% of students overall selected 21–30 oocytes, while only 25% answered correctly that 40+ oocytes were required [11].

## Discussion

As elective oocyte cryopreservation takes increasing prominence in society and medicine, we sought to compare responses between medical students and undergraduates, who as groups differed significantly in age, race, and religion (Table 1). Further, undergraduates vary in their exposure to and knowledge of reproductive medicine.

Most undergraduate and medical students would not consider elective oocyte preservation at the present time. This is in line with previous studies, where the cost of oocyte preservation is the limiting factor in younger women’s ability to undergo the procedure [10, 12]. Even though the majority of both groups would not undergo oocyte cryopreservation, undergraduates were more price-sensitive at the current price. This might be influenced by medical students’ perception of future earning potential, or desire to preserve their fertility in a long educational pathway.

A significant majority of women in our study would be more likely to consider elective oocyte cryopreservation if their employer paid for it. Well-known companies have begun a trend toward this in the past few years. According to media reports, starting January 1, 2015, Apple™ was willing to pay up to $20,000 per employee for oocyte cryopreservation and storage for all full-time and part-time employees. Facebook™ introduced a similar benefit for its employees in the United States in January 2014 and pays up to $20,000 for both medical and non-medical oocyte cryopreservation [13, 14].

However, oocyte preservation is not a guarantee of future fertility. Donor egg studies show that age of the oocyte donor is a significant predictor of pregnancy success and is a major factor in selecting prospective candidates [15]. There may be also deficiencies created by the freezing process itself [5]. In 2015 the pregnancy rate was 52.7% for frozen donor egg transfer in comparison to 65.9% for fresh donor egg transfer [16].

In our study medical students favored undergoing cryopreservation at ages 31–35 while most undergraduates would undergo it at 26–30. This may have reflected the differing age of each group, where each group thought they would consider freezing about 5–10 years in the future. However, literature shows that even in younger women (i.e., < 38 years old), the clinical pregnancy rate (CPR) per thawed oocyte is low, ranging from only 4.5–12% [17] and the likelihood of success decreases with age at freezing. To have a greater than 50% chance of live birth at age < 35, a woman must freeze at least 10 oocytes. At age 40, this number increases to 40 oocytes [11]. Women in our study consistently underestimated this number, and only 25% of women selected the correct number of eggs.

Oocyte preservation may be a reasonably effective method of fertility preservation if adequate numbers of eggs are frozen at a younger age, but due to the expense, cost-effectiveness is important to discuss when translating these findings to patient counseling. The cost-effectiveness assessment adds the question of whether frozen oocytes would actually be used. Cobo et al. reported that of 1469 patients undergoing oocyte cryopreservation, only 137 (9.3%) returned to use the frozen oocytes at a mean age of 39.2 (95% CI 39.0–40.1) [18]. The most cost-effective strategy varies depending on age of desired childbirth. Indeed, at a relatively younger age, a woman would be likely to become pregnant on her own and would not require frozen oocytes. Due to this, some suggest the most cost-effective strategy is to encourage woman to not delay childbearing [19]. Following from this reasoning Mesen et al. find that over a 7 year horizon oocyte cryopreservation provides the greatest improvement in probability of live birth compared with no action when performed at age 37 years (51.6% vs. 21.9). [4]. However, in absolute terms, the younger the age of egg freezing, the more successful the pregnancy outcome [20].

Assuming a woman will desire pregnancy at an older age where natural conception is less successful, an important question is whether undergoing an IVF cycle at that age is more cost-effective than using frozen oocytes from her younger age. A model developed by Devine et al. assuming pregnancy attempt at age 40 showed that the most cost-effective approach was attempting spontaneous conception by intercourse for 6 months then using oocytes that were frozen by age 35. Using frozen oocytes was more cost effective than attempting IVF at age 40 for two cycles. When age of oocyte cryopreservation was varied, the approach using frozen oocytes instead of undergoing fresh IVF remained more cost effective up to age 38. After this age, it was not cost-effective to freeze oocytes [21].

When asked about the age at which fertility significantly decreases, a large proportion of undergraduates, 35%, believed that this occurred at age 41–45. The majority of medical students believed this occurred at a more realistic 36–40. However, in reality, fecundity begins to decrease at age 30; in addition, it has been demonstrated that the chance of not conceiving a first child within 1 year increases approximately 6-fold when women over 30 years of age [20]. In recent decades, numerous reports have confirmed that the probability of a live birth decreases distinctly after the age of 35, especially with ART. [20]

This overly optimistic impression of late and very late pregnancy success may be a result, at least partially, from media coverage of such pregnancies. In an analysis of British media portrayals of older mothers, a preponderance of coverage relating to celebrities was identified. Delayed childbearing was represented positively, as part of a life plan allowing women to have the “best of both worlds,” and the media did not acknowledge age as an obstacle to pregnancy [22]. A study on public perception showed that advanced maternal age was not perceived as a risk factor and that the increased risks and complications were not elaborated on by the media [23].

Our study has several limitations. Our population size is small but included a mixture of science undergraduates, non-science undergraduates, and medical students. We were able to compare students at different points in their lives and education to understand similarities and differences in perspectives. However, we were limited to students and our findings may not generalize to the population not pursuing formal education. Further, our study was limited to a local population in Eastern Virginia and a larger nationwide survey to undergraduates and medical student would better represent the population trend.

## Conclusion

Most undergraduate and medical students would not undergo elective oocyte cryopreservation at the present cost. However, a significant majority of women in our study would be more likely to consider it at a lower cost or if their employer paid for it. We identified a need for education on age-related changes in fertility in both undergraduates and medical students, as students tended to overestimate the age of fertility decline. This may suggest a wider need for public education, particularly regarding efficacy of cryopreservation with increasing age, cost-effectiveness of cryopreservation, and age-related fertility decline.

## Additional file

Additional file 1: Oocyte Cryopreservation (egg freezing) Survey. (DOCX 17 kb)

## Abbreviations

ART: assisted reproductive technology
MS: medical students
UG: undergraduate students
