# Supplementary material for: Knowledge and attitudes regarding elective oocyte cryopreservation in undergraduate and medical students
Source: Fertil Res Pract. 2019 Apr 10;5:5. doi: 10.1186/s40738-019-0057-9 (PMC6458751; doi:10.1186/s40738-019-0057-9)
Supplement: Supplementary file 1 — Oocyte Cryopreservation (egg freezing) Survey. (DOCX 17 kb) [file 40738_2019_57_MOESM1_ESM.docx]

**Oocyte Cryopreservation (egg freezing) Survey**

1. What is your race?

- Caucasian / White
- Hispanic / Latino
- African American
- Asian
- Other

1. What is your age? _______
2. What is your religion?

- Buddhist
- Muslim
- Christian
- Hinduism
- Catholic
- Non-religious
- Other

1. If you are currently in school, what level of student are you?

- Undergraduate
- Medical student

1. Do you have children or are you currently pregnant?

- Yes
- No

If so, at what age did you have your first child?

- 21–25
- 26–30
- 31–35
- 36–39
- >40

If not, do you plan to have children?

- Yes
- No

If yes, what age do you plan to have your first child?

- 21–25
- 26–30
- 31–35
- 36–39
- >40
- Not sure

1. Do you face pressure from your family to have children?

- Yes
- No

1. What is your level of knowledge about oocyte cryopreservation (egg freezing)?

- None
- Some knowledge
- Moderate knowledge
- Extremely knowledgeable

1. At what age would you consider oocyte freezing?

- 21–25
- 26–30
- 31–35
- 36–39
- >40

1. For what reasons would you consider oocyte cryopreservation? (You may choose more than one)

- Medical (ex. while undergoing radiation therapy)
- Social (ex. no current partner)
- Career (ex. education, career prior to children)

1. Do you think this procedure has a negative effect on your future fertility?

- Yes
- No

1. Currently at approximately $10,000, would you consider oocyte cryopreservation?

- Yes
- No
- At a lower price

1. Who should pay for the oocytes freezing?

- Self
- Parents
- Insurance
- Employer/Company

1. If your employer paid for oocyte freezing, would you be more amendable to freezing your eggs?

- Yes
- No

1. At what age do you think fertility *significantly* decreases?

- 30-34
- 35-39
- 40-44
- 45-50

1. At what age do you think it is too old to have a child naturally?

- 30-34
- 35-39
- 40-44
- 45-50

1. When deciding on oocyte cryopreservation, which of the following would impact your decision? (You may choose more than one)

- *Decreased infertility with age (typo in survey so question discarded)*
- Increase in miscarriages with age
- Increased chance of having a child with Down Syndrome with age

1. What do you think is the minimum number of eggs you need to freeze at age 30 to make it likely to have a live birth using them in the future?

- 5-9
- 10-19
- 20-29
- 30-39
- 40+

1. What do you think is the minimum number of eggs you need to freeze at age 40 to make it likely to have a live birth using them in the future?

- 5-9
- 10-19
- 20-29
- 30-39
- 40+
